# Psychiatric poetics: mental healthcare and Giovanni Stanghellini's ‘Logics of Discovery’

**DOI:** 10.1192/bjb.2024.115

**Published:** 2026-02

**Authors:** George Ikkos, Alastair Morgan

**Affiliations:** 1 Department of Psychiatry, Royal National Orthopaedic Hospital, London, UK.; 2 University of Manchester, Manchester, UK.

**Keywords:** Schizophrenia, Aby Warburg, Friedrich Hölderlin, psychiatric interview skills, patient experience and co-creation

## Abstract

The importance of art and humanities in mental health is widely recognised, and consumption and creation of poetry, prose, drama and the plastic arts are now considered to be relevant knowledge-generating and therapeutic activities. However, literary and art criticism remain at the margins. By contrast, in his two ‘Logics of Discovery’ papers, psychiatrist, psychopathologist and psychotherapist Giovanni Stanghellini brings to bear on clinical discovery and the healing alliance cultural historian Aby Warburg's approach to images (specifically, his *Atlas of Mnemosyne*) and philosopher Giorgio Agamben's analysis of the linguistic phenomenon of parataxis in Friedrich Hölderlin's poetry. Both Warburg and Hölderlin experienced severe mental disorders, and Stanghellini's analysis is notable for its potential to contribute to co-creation in a wide range of clinical settings. We suggest that this work may help to address some key sources of dissatisfaction among mental health patients and thus improve patient experience and clinical outcomes. We also comment on issues regarding implementation of Stanghellini's proposals and conclude with discussion of an example of the severe loosening of associations originally reported by Eugen Bleuler.

‘A constant trait of poetry, throughout its history, is some elusive *(je ne sais quelle*) affinity with mystery but not at all with the inexpressible (*l'indicible*) as people too frequently claim. Poetry rather involves a sense of propriety, a voluntary reticence, a certain way of hushing what is essential, in order to merely lure the imagination, to let it develop the message as it might wish. Behind the letter of the message, something unexpressed seems to await the moment of delivering a secret- a secret known from the outset and which had to be roused.’ (Roger Caillois, Le Fleuve Alphée^1^)

‘I am creating a language which must necessarily spring from a quite new conception of poetry, and I define it in these words: To paint, not the thing, but the effect which it produces.’ (Stéphane Mallarmé in a letter to Henri Cazalis, 30 Oct 1864^2^)

‘… with lime-twigs you can catch birds – never catch their birdsong’ (Odysseus Elytis^3^).

Whether founded in biomedicine, academic psychology, psychodynamic theory, or some other social, philosophical or spiritual therapeutic narrative, theories of mental health and illness obscure clinical phenomena to some extent, some more and others less, but precisely through the constraints their specific narratives impose. Arguably, allowing phenomena to show themselves in a manner akin to that suggested by Caillois and Mallarmé could overcome such limitations and allow us to catch them in full song.

Etymologically, the word ‘poetic’ arises from the Greek *ποιɛῖν*, which means ‘to make’. In their *Autopoiesis and Cognition*, Chilean biologists Humberto Maturana and Francisco Varela first proposed that each living organism is an ‘autopoietic machine’, one that constantly constitutes and realises itself as a concrete unity in space through a network of internal interactions.^4^ All life is fundamentally organised around poetic processes of production, transformation, regeneration, destruction, interaction and realisation (and decay). In addition, human life is poetic in the sense of ‘making’ things, also more abstractly poetic in ‘making sense’ of things.

In relation to psychological aspects of experience and healing, it is Giovanni Stanghellini's thesis that current biomedical, psychological and psychodynamic approaches, owing to the constraints their theoretical narratives mandate, fail to take fully on board the (auto)poetic nature of patients and providers. In seeking to redress this shortfall, first, in *The Power of Images and the Logics of Discovery in Psychiatric Care* (Logics of Discovery I), he takes his cue from art critic Aby Warburg's *Atlas of Mnemosyne*, a collection of images through which Warburg shed new light on Florentine Renaissance art;^5^ and, second, in *Lessons from Poetry – Parataxis as a Method That Can Complement the Narrative Compulsion in Vogue in Contemporary Mental Health Care* (Logics of Discovery II), he does so from philosopher Giorgio Agamben's linguistic analysis of the phenomenon of ‘parataxis’ in Friedrich Hölderlin's poetry.^6^

Stanghellini's clinical innovation is that in attending to images and parataxis, he seeks to study the ‘syntax’ of the therapeutic dialogue. This syntax is not concerned with narrative unity but focuses on its gaps. Through these, in an encounter where space is held open for diverse forms of sense, different shapes of meaning may emerge that can be both abstract and concrete, both fragmentary and more fully formed. This way of attending to the patient is particularly sensitive to the fragmentary as it appears in language and in images.

In this paper, we first summarise Stanghellini's two papers, before discussing their significance in terms of everyday clinical practice, including when working with severely distressing mental states. We suggest that there is an imperative to incorporate their insights in training and practice because they offer a new logic of discovery that facilitates co-creation in a healing encounter. Such co-creation attends to a different syntax of the encounter, following fragments and gaps in meaning and experience to improve both patient and therapy experience and enhance outcomes.

## The power of images and the Logics of Discovery in psychiatric care

In Logics of Discovery I, Stanghellini identifies three very general patterns through which psychiatrists and other mental health practitioners may approach and try to make sense of patients: ticking boxes, drafting arrows and linking dots.

The paradigmatic tick-box approach in contemporary mental healthcare is the American Psychiatric Association's succession of Diagnostic and Statistical Manuals. The clinical assessment process here consists of ascertaining the presence or absence of specific symptoms and signs and ticking diagnostic criteria accordingly. The validity of psychiatric diagnosis according to this approach is now widely disputed,^7^ and, as Stanghellini emphasises, it fails to generate a genuine process of discovery. Rather, it is like two people sitting in front of a puzzle, with the clinician having possession of the master picture and the patient's predetermined and strictly limited number of pieces expected to fit in. Stanghellini's key criticism of both the ticking boxes and drawing arrows approaches is that because of their focus on objectivity, which is scientific at times and scientistic at others, they fail to engage with the fundamentally emergent nature of mental phenomena.

Both biomedical and psychodynamic approaches are considered by Stanghellini as exemplars of the drafting arrows approach. In academic psychology, such an approach is often represented by drafting arrows between boxes. A key feature here is the assumption of linear causation. Owing to this and other features, all these approaches risk reductionism. Stanghellini observes that whereas sweeping reductionism has retreated in recent years, ‘creeping reductionism’ remains a reality. He states specifically that this ‘involves partial reductions that work on a patch of science, where bit by bit we obtain fragmentary explanations using disparate interlevel mechanisms including neurotransmitters, genes, neural circuits, etc. Creeping reductionism seems to tolerate a kind of pragmatic and pluralistic parallelism’. He offers a focus on ‘linking dots’ and ‘emergentism’ as a complementary and in some ways more appropriate approach.

Both approaches of ‘ticking boxes’ and ‘drafting arrows’ attempt to trace back the experiences of severe mental illness and thought disorder to an underlying, determining factor, whether psychological or biological. In doing so, they miss something about the fragmentary experience that they attempt to define. By contrast, the approach of ‘linking dots’ that Stanghellini outlines draws attention to experiences that cannot be linked in a chain but stand out as an ‘exception, a singularity, that arouses perplexity and disrupts the previously acquired representation of the patient's condition.’

### Linking dots: emergentism and Aby Warburg's *Atlas of Mnemosyne*

In the same paper, Stanghellini states that ‘Although there is no single and neat concept of emergence independent of particular explanatory contexts, an emergent phenomenon can be roughly defined as a phenomenon that is irreducibly relational; emergent features are not reducible to any intrinsic property of any element of a whole’. Relational phenomena can be mediated and expressed through intrapersonal, interpersonal and sociocultural processes. For example, it is important to attend not only to any hypothesised or confirmed biological process that may be related to specific symptoms but to the patient's own response to this, which is also crucial to understanding and acting therapeutically in the clinic. He emphasises the importance of the observer, the observed, the context of observation and their interactions in determining the specifics of the emergent phenomena. He highlights the circularity of processes and illustrates this by attending to Aby Warburg and his *Bilderatlas Mnemosyne* (*Atlas of Mnemosyne*; *Memory's Atlas*; https://warburg.sas.ac.uk/archive/bilderatlas-mnemosyne/final-version).

Aby Moritz Warburg (1866–1929) was the scion of a highly successful Jewish banking family in Hamburg. The headquarters of the oldest surviving bank, which remains privately owned, have remained continuously in Hamburg since its foundation in 1798, but Aby Warburg forewent his inheritance in favour of his brother. His only condition was that his brother would secure for him any book he required. His book collection is the foundation of the Warburg Institute (https://warburg.sas.ac.uk/), which moved during the Nazi era and is now housed in London. Warburg attended two terms of medical school but graduated in the humanities in Bonn and is best known as a pioneering art historian. He suffered from mental illness and spent the years 1919–1924 in Ludwig Binswanger's Bellevue Clinic at Kreuzlingen, Switzerland; there, he was examined by Emil Kraepelin, who reached the diagnosis of schizoaffective disorder.^8^ It was while resident in Bellevue that he produced *Atlas of Mnemosyne*, partly as a therapeutic measure for his own condition. Although he retained his professorial post in the History of Art in Hamburg after discharge, his academic activities remained limited thereafter.

Warburg's key academic achievements include his challenge to the ‘great artist’ view of the history of art. *Atlas of Mnemosyne* remained unfinished at the end of his life; at that time, it consisted of 63 wooden panels covered with black cloth, on which were pinned about 1000 pictures from books, magazines, newspapers and other daily life sources (https://warburg.sas.ac.uk/aby-warburg-bilderatlas-mnemosyne-virtual-exhibition). Via contemplation of these images, which were disparate both in nature and quality, he sought to identify correspondences emerging out of their juxtaposition and the affective bundles (*Pathosformeln)* they generate in the observer. These served him as a guide to the key evolving patterns in the history of Renaissance art.

Stanghellini thinks of ‘dots’ in his model as fragments of experience that function as images do in Warburg's approach to his wooden panels. These fragmentary images can be ‘thought of as pictures to hang on a wall’, pictures that generate affective responses and resonate in correspondence with each other without being joined together by any causal or narrative logic. Stanghellini aims to bring over to mental health practice Warburg's highly educated but deliberately conceptually unformulated approach to the objects of his study and his attention to affect, together with his tolerance and even welcome of fragments of impressions that reach the observer. It is his attention to fragments as well as fuller patterns of experience, perception and feeling that makes his approach relevant to severely disturbed mental states.

## Lessons from Poetry – Parataxis as a Method That Can Complement the Narrative Compulsion in Vogue in Contemporary Mental Health Care

In Logics of Discovery II, Stanghellini challenges the impulsion towards narrative in mental health practice by discussing loosening of associations, loss and discovery of meaning, psychopathology and poetry from the perspective of *parataxis*. He defines parataxis as ‘the bracketing of connections between the parts of a discourse, letting them float rather than be immovably grounded in pre-established narrative relations’. Referring to both psychopathology and poetry, he leaves a question mark over the relative importance of compulsion and choice in this loosening or parataxis. He allows that compulsion may be stronger in psychopathology and choice greater in poetry but explores the overlaps between the two. Loosening of associations and parataxis occur both in psychopathology and literature.

‘At the time of the New-Moon Venus stands in the August heavens of Egypt and with its rays of light illuminates the harbors of commerce, Suez, Cairo, and Alexandria. In this historically famous city of the Kalifs, there is situated in the museum of Assyrian monuments from Macedonia. There plantain flourishes next to maize columns, oats, clover, and barley, also bananas, figs, lemons, oranges, and olives. Olive-oil is an Arabian liqueur-sauce, with which Afghans, Moors, and Moslemites carry on the breeding of ostriches. The Indian plantain is the whiskey of the Parsee and of the Arab. The Parsee or the Caucasian possesses exactly as much influence over his elephant as the Moor has over his dromedary. The camel is the sport of the Jew and the Indian. In India, barley, rice, and sugar-cane, that is, artichoke, flourish luxuriantly. The Brahmins live in castes on Beluchistan. The Circassian inhabit Manchuria of China. China is the Eldorado of Pawnees.’

Stanghellini shares the above quote from Bleuler to illustrate a rather extreme form of loosening of associations. He then segues to the work of Klaus Conrad^9^ and the latter's observation that the ‘cloud’ of such fragmented details in severe psychopathology sometimes evolves and suddenly hangs together. This way, new meaning comes to be perceived. However, this meaning does not evolve from syllogisms utilising *hypotaxis*. Rather, Stanghellini suggests, the phenomenon of parataxis sometimes can clear the ground from habitual connections to allow the emergence of unexpected new ones. Stanghellini writes:

‘In rhetoric, parataxis (from Greek: παράταξις, “act of placing side by side”; from παρα, para “beside” + τάξις, táxis “arrangement”) refers to the practice of placing phrases or parts of speech next to each other without subordinating conjunctions. Parataxis, in writing or speaking, favours short, simple sentences. It contrasts with hypotaxis (from Ancient Greek: ὐπό, hypò, “under” and τάξις, táxis, “arrangement”), the syntactic structuring whereby the period is characterised by different levels of subordination.’

Examples of paratactic writing abound in modernist and contemporary literature. Hölderlin (1770–1843) is important because through his poetry, he was the first to write this way, and he was probably meeting diagnostic criteria for schizophrenia at the time of such writing.^10^ Stanghellini suggests that such practice may be understood as expressing specific forms of perception, thought or language or a combination. He quotes what is generally considered to be an outstanding example of parataxis, written in Hölderlin's exact midlife when signs of his illness started becoming clearer:

‘With yellow pears hangs –
And full of wild roses –
Land hangs into the lake,
O lovely swans,
And drunk with kisses
You dip your heads
Into the sacred-sober water.
Alas, where shall I find,
When winter comes, flowers, and where
The sunlight,
And the shadows of Earth?
The walls stand
Speechless and cold; in the wind
Weathervanes clatter’

There is greater coherence here than in Bleuler's quote, but the key point is that two images, one of light and plenitude and the other of winter darkness and want, are juxtaposed without resolution. In its non-resolution, parataxis is decidedly not dialectical in the narrow or reduced sense of dialectics, which demands that thesis and antithesis always lead to a new synthesis. Rather, in a broader sense, we find here dialectics without forced resolution. Images are left suspended in their contradiction in order to produce new correspondences of meaning.

French poet Charles Baudelaire (1821–1867), another brilliant and troubled man, with a history of self-harm and addiction in his case, was a master practitioner of parataxis in the 19th century. As a different way to knowledge, Baudelaire offers analogy instead of synthetic syllogism and drafting arrows. Analogies require identification of correspondences between phenomena which cannot be logically deduced. Stanghellini quotes the German polymath and writer Johann Wolfgang von Goethe (1749–1832): ‘If one follows analogy too closely, everything coincides in the identical; if one avoids it, everything disperses into infinity.’

Here, we can see the fragility of these experiences of thought disorder that hover above a groundlessness of meaning. This groundlessness can certainly produce a fragmentation of experience. Stanghellini writes of experiences of itemisation in severe mental illness, the way that objects fragment and stand out, and the captivation of attention by small details. However, attention to poetic parataxis offers a different way of understanding this fragmentation as an opening to new possibilities of meaning. This is not to affirm either a vision of mental illness as fragmentation or as creative production but to hold both these experiences together in an attempt to understand emergent meaning. Stanghellini writes of navigating ‘an intermediate zone between mysticism and psychopathology, aware that this is a narrow strip, at whose edges the waters necessarily mingle’.

Sips has written of this intermediate zone as a dialectic of ‘aha’ and ‘anti-aha’ experiences, drawing on Conrad's work. In the aha experience, there is a sudden emergence of correspondences, where everything just hangs together in a revelation of essential meaning; however, this experience can easily tip over into the anti-aha experience, an experience of loss of all meaning that undermines all that went before.^11^ In their qualitative study of these experiences in psychosis, Sips et al.^12^ outline the way that the sudden insight or aha experience produces a radical revelation of truth through forging associations and connections, but that this process takes on a life of its own, and an altered sense of reality spreads to a frightening loss of grounding in the world. Sips et al. note that a primary clinical focus on overt symptomology in psychosis, namely hallucinations and delusions, often misses important aspects of psychotic experience, such as Bleuler's original focus on the fragmentation of processes of thinking and experiencing.

## Discussion

A compulsion to create narrative meanings often forces strange experiences into a mould where it is assumed they must be normalised. From this perspective, sense can only be made of such experiences by making them metaphors for some deep underlying conflict or traumatic event. Everything must make sense within this ‘narrativistic compulsion’, and that sense is one that dissolves the experiences of mental illness as it explains them. From this perspective, once the deep underlying factor (whether biological or psychological) is discovered, the strange experiences that drove the explanatory process are dissolved as epiphenomena. By contrast, Stanghellini emphasises an open encounter that dwells with anomalous experiences to try to produce an emergent meaning that is not determined in advance. Patients have reported the importance of such approaches to acknowledging uncertainty and contingency within strange experiences and the centrality of a listening which refuses to impose too rapidly a narrative meaning.^13^

Clinical practice in mental health is both a science and an art. Secure clinical knowledge can be attempted both through the findings of positive natural science and the analogies to be found in humanities and the arts. In truth, extreme box-ticking aside, the clinical encounter has always incorporated elements of both. However, whether the approach has been biological, behavioural or psychodynamic and even under the rubric of the biopsychosocial model, there has always been a dominating, even intrusive narrative. Stanghellini offers new building blocks that both stand in tension with and complement established approaches. This is because of the attentiveness he urges towards elemental phenomena, namely images and parataxis, emerging within the encounter. Eschewing any theoretical assumptions or predetermination, these have the potential to allow for the emergence of new meaning.

In truth, without negating the above, as Stanghellini himself points out, his approach has affinities with Freud's method of free association. He highlights Freud's own insistence on the analytic as opposed to synthetic imperative in psychoanalysis and Freud's confidence that as in ‘chemical reactions’, the emergent elements will synthesise themselves into new, sometimes unforeseen constellations. Where Stanghellini differs, however, is in the distance he puts between his approach and the whole narrative that is psychoanalytic theory. No doubt, psychoanalysis continues to develop and remains relevant to a wide range of mental health practice, but it is the elementality of the images and parataxis that Stanghellini highlights that promise their wider applicability. Unlike psychoanalysis, which arose from and continues to focus on patients with dissociative and common mental disorders, images and parataxis emerge from the critical and poetic practice of two men with severe mental disorders. The juxtaposition without synthesis that Stanghellini invites us to attend to offers a way to engage and value even fragments of experience and, without theoretical baggage, have the opportunity to co-create meaning or even tolerate the infinite burden of lack of meaning together with patients. This is a form of psychotherapy that operates under privation, an abstinence from ‘ordinary interpretations and narrativistic constructions’.^6^

The conceptual simplicity of images and parataxis, which makes them particularly valuable when dealing with severely disturbed mental states and the countertransference they evoke, suggests that they will be relatively easy to convey intuitively to trainees, even as the utility of this simplicity will increase with their experience.^14,15^ Nevertheless, it may be objected that in the already crowded space and/or time of the consultation mandated by hard-pressed services and managerial performance requirements, it is difficult to see where these modest proposals may fit in. In the face of widespread patient dissatisfaction with mental healthcare, however, can we afford any longer to ignore such sources of discovery and co-production of meaning?^16^ On the contrary, we would suggest that their inclusion will be essential to any future humanisation of the culture of mental health services and care.

## Conclusion

‘Whoever cannot seek the unforeseen sees nothing, for the known way is an impasse’ (Heraclitus, quoted in ^17^). A central challenge for contemporary psychiatry and mental health services is co-production. Stanghellini offers foundations to achieve this by providing a sensitive framework for the facilitation, co-creation and recognition of emergent meaning. This includes attention to elements and fragments of life histories that do not necessarily cohere easily into a singular narrative. As is apt when considering poetry, speaking metaphorically, we may say that he has discovered new elements in the ‘periodic table’ of the chemistry that makes up the clinical consultation and psychiatric treatment process – or, alternatively, that he has added new genes to the polynucleotide chain of the specialty's DNA. His work shares with these the creativity arising from attention to elementary processes of difference and repetition; in his preferred words, it can enrich the syntax of our consultations.

In their simplicity as concepts, images and parataxis offer potential for repeated use during clinical encounters, not unlike the routine act of prescribing. Like prescribing, they are the product of extended background scholarly and scientific investigation, and their successful use will depend on clinical sensitivity and discretion. This being so, it is then a matter of further empirical enquiry to establish whether, as we believe, this practice will improve the experience of care, patient-reported outcomes and quality of life, even possibly symptomatic control. If these benefits materialise, reasoning by analogy again, we may have to ask whether art and literary criticism, ‘basic sciences’ to the humanities, may need to be added to those of psychiatry, to complement genetics, epidemiology, phenomenology and so on.

It may take some time until we reach the above state of affairs, though perhaps less than may be thought by many.^18^ In the meantime, today's psychiatrists may wish to return with us to the quote from Bleuler. Although Stanghellini does not say so, if we set aside any usual demands for narratives and hypotaxis, it is difficult not to think that there is an overall reference to something, possibly the European imperial imaginary, with a taste for the exotic. Or it may be something different that the patient's words communicate, perhaps that he is on some bewildering journey. And if we allow our imagination to stay with some of the images and the elementary sensations, such as the smells of fruits and spices that the patient's words can evoke, we may hit on something more precise which has special resonance for him. Any of these speculations may or may not be true, but they may offer an opportunity to share or begin to discover or co-create meaning with the patient. And, if the endeavour is unsuccessful, we may have a better sense of the distance that separates us and the patient's particular humanity. Although some, including Freud, have thought that patients with schizophrenia do not care about relationships, they certainly do, and they do so in their own way.^10,19,20^ Crucially, as Henri Poincaré noted, even in that most rigorous of discipline, mathematics, some of the most important work is not done with deduction and hypotaxis:

‘[creative] mathematical work is not simply mechanical … it could not be done by a machine, however perfect. It is not merely a question of applying rules, of making the most combinations possible according to certain fixed laws. The combinations so obtained would be exceedingly numerous, useless and cumbersome. The true work of the inventor consists in choosing among these combinations so as to eliminate the useless ones or rather to avoid the trouble of making them, and the rules which must guide this choice are extremely fine and delicate. It is almost impossible to state them precisely; they are felt rather than formulated … the subliminal self is in no way inferior to the conscious self; it is not purely automatic; it is capable of discernment; it has tact, delicacy; it knows how to choose, to divine. What do I say? It knows better how to divine that the conscious self, since it succeeds where that has failed. In a word, is not the subliminal self superior to the conscious self?’^21^

## About the authors

**George Ikkos** is a consultant liaison psychiatrist in the Department of Psychiatry at the Royal National Orthopaedic Hospital, London, UK; immediate past Chair of the Royal College of Psychiatrists’ History of Psychiatry Special Interest Group; and former President of the Royal Society of Medicine's Pain Medicine and Psychiatry Sections. He is joint editor of *Mind, State and Society: Social History of Psychiatry and Mental Health in Britain 1960–2010*, which was runner-up (Silver award) in the Association of American Publishers’ PROSE 2022 Awards, in the section History of Science, Medicine and Technology (available open access from https://doi.org/10.1017/9781911623793). **Alastair Morgan** is a lecturer in Mental Health Nursing at the University of Manchester, UK. His most recent book is *Continental Philosophy of Psychiatry. The Lure of Madness*, published by Palgrave MacMillan in 2022 (https://link.springer.com/book/10.1007/978-3-031-09334-0).
